# Urinary Diseases and Their Treatment

**Published:** 1839-01

**Authors:** 


### ART. VIII.

*Urinary Diseases and their Treatment.* By ROBERT WILLIS, M.D., Physician to the Royal Infirmary for Children, &c.—*London*, 1838. 8vo. pp. 408.

THERE have been, of late years, since the examination of the urine has been conducted on principles tolerably well understood, many valuable contributions on the subject of urinary diseases. In our own country, Marcet, Bostock, Brodie, Prout, &c. may be mentioned, as having acquired much celebrity, in this department of medicine, from the light thrown by their researches on matters previously very little known, or involved in much obscurity. On the continent, there have been many contributors to the same subject. The communications to the public, of the discoveries of these various experimenters, lie in individual volumes, in Transactions of Societies, in Journals, &c.; and it was a matter very much to be desired, that some one competent to the task should trouble himself with the making of a compilation, which should contain a comprehensive arrangement of all that was known.

We do not know that a more competent author than Dr. Willis could have been found to undertake the task: possessing, as it is evident from his work that he does possess, an accurate acquaintance with the subject in all its details, considerable personal experience in the diseases of which he treats, capacity for lucid arrangement, and a style of communication commendable in every respect. His object has been "to give a comprehensive and connected view of the functional derangements of the organ which secrete and excrete the urine, and of the medical and dietetic treatment adapted to their different forms;" and "a particular consideration of stone in the kidney and bladder, and of its remedy by medical means." Connected with the descriptions of various conditions of urine are modes of analysis, sufficiently delicate for common purposes, some of which will be very acceptable to many of our readers. From the character of the work, our notice of it must be expected to be very incomplete; although, if our estimate of its value could only be judged of by the length of our review, we should scarcely know where to stop.

Its introductory chapter treats of the physiology of the kidneys, and of the natural constitution of the urine. Especial reference is made to these organs as excretors of nitrogen; the importance of which office is not only evinced by morbid phenomena, but by the very general existence of the kidneys, or of organs allied to them in function, throughout the animal kingdom. It is remarked that the quantity of nitrogen in the food is the chief element in determining the amount of solid excrementitious urinary matters. It is well known that the kidneys are both *secreting* and *separating* organs, urea having been detected in the blood of animals whose kidneys had been extirpated; but the exact limits of these two functions cannot be at all determined. Dr. Willis thinks that the average specific gravity of healthy urine, among grown individuals, cannot be estimated more closely than 1·015. Whilst on this subject, we would refer to the table of Dr. Bostock, published in the last volume of the Medico-Chirurgical Transactions, noticed in a former article; and we would also insist on the necessity of employing instruments, in ascertaining the specific gravity of the urine, the scales of which accurately correspond: we have seen urinometers prepared by different makers, enjoying a good reputation, the scales of which were entirely discordant. Elliott, 112, High Holborn, is the maker of the urinometer which Dr. Willis employs.

The following is Dr. Willis's arrangement of his subject: The first part of the book is devoted to functional derangement of the kidneys and their immediate consequences; the second to functional derangement of the organs which excrete the urine. The first part contains eight chapters; the titles of which, to prevent recapitulation, and to put our readers in possession of the general scope of the volume, we here extract:

"Chapter 1. Morbid states in which the secreting faculty of the kidneys is exalted, and the menstruum and readily-soluble principles of healthy urine occur in altered absolute or relative quantity. Ch. 2. Morbid states in which the secreting faculty of the kidneys is lessened or abolished. Ch. 3. Morbid states in which the urine contains, in excess and as precipitates, certain ingredients that occur normally in smaller quantity and in solution. Ch. 4. Morbid states in which the urine contains, in solution or as precipitates, certain principles which do not occur in the healthy secretion, but appear to be derived immediately from one or other of these. Ch. 5. Morbid states in which certain matters being constituents of the blood are contained in the urine. Ch. 6. Morbid states in which the constituents of secretions and altered elements of the blood are discharged with the urine. Ch. 7. Morbid states in which principles foreign to the urine and the blood, and derived from none of the natural constituents of these fluids, are eliminated by the kidney. Ch. 8. Consequences of one or other of the morbid states described, particularly of those comprised in Ch. 3 and 4.—Urolithiasis, the formation and growth of urinary calculi."

Regarding the above as an entirely artificial arrangement, we can see no objection to it; but the nomenclature applied to the subdivisions or sections, we think is the most defective part of Dr. Willis's book. There are abundant objections to many terms at present applied to urinary diseases, and a correct system of nomenclature is a great desideratum. But, to substitute a new inappropriate designation for another, is a very great defect. We will illustrate what we mean. The first part of the work before us treats of "functional derangements of the kidneys and *their immediate consequences*," and beneath the first chapter we find the following Sections. "Sect. 1. Of the discharge of urine, which is characterized by a deficiency of solid matters, generally, *Hydruria.* Sect. 2. Of the discharge of urine which is characterized by a deficiency of urea, *Anazoturia.* Sect. 3. Of the discharge of urine which is characterized by a superabundance of urea, *Azoturia.*" Now, had these terms "Hydruria, Anazoturia, Azoturia," been applied merely to certain conditions of urine, they might have been free from objection, provided that they were descriptive of the condition, and included the whole morbid change. But the heading of this first part shows us that these terms are intended to apply to functional derangements of the kidneys and *their immediate consequences*; and under each section is described a disease of which the condition of the urine is but one of the symptoms: so that the term debility, emaciation, or any other attendant condition might with as much propriety be employed as a designation, as those which have been adopted, from one condition of the urine alone. If we look at the function of the kidney to be not only secreting, but separating, that is, acting merely as a sieve upon principles existing in the blood, the impropriety of the above nomenclature is more manifest; for, in what part of the system does the fault commence? It may be in the digestive apparatus: it may be originally in the blood: it may be in the absorbent system: it may be in one or more, or in none of these. If doubts of this kind are admitted to exist, there can be no question of the impropriety of introducing any new names of the character of those which we have noticed. At the same time, however, that we make these observations, we must say, that in none of his chapters does Dr. Willis appear to have taken other than a comprehensive view of his subject.

Under the term *Hydruria*, by which is meant a discharge of urine characterized by deficiency of solid matters, generally, a condition is referred to as existing in advanced years which has not met with the attention which it deserves. There are frequent calls to make water, connected with *an augmented flow of urine of low specific gravity;* the case generally being regarded as one of irritable bladder, the quality and quantity of the urine being overlooked. The indications for treatment which are mentioned are sufficiently obvious, consisting mainly in attention to the general health. In the aged, laudanum is regarded as likely to produce very beneficial effects.

The next section treats of the discharge of urine which is characterized by a deficiency of urea, *Anazoturia.* Here the urea is both relatively and positively deficient. This, as well as the former disease, has been described as *diabetes insipidus*, and Dr. Willis believes that many of those cases of diabetes which have been described as cured have belonged to this head. The only cases of it which he has seen had been in children. The urine is very abundant, limpid, of a pale straw colour, or colourless, and of a very faint smell. It is weakly acid or neutral when first voided, rarely precipitates within twenty-four hours, but soon changes; it becomes faintly ammoniacal and covered with a fine creamy pellicle on its surface, which shows the minute pearly-white crystals of the ammonia-magnesian phosphate. The cases seen by Dr. Willis, with other instances collected from books, and classed with much probability with them "have many features in common. They were all characterized by thirst, gnawing sensations at the pit of the stomach, white furred tongue, constipated bowels, and a parched state of the skin. There was also more or less of emaciation, considerable loss of strength, and generally great depression of spirits in all. The hyperuresis in this class of cases, is, therefore, a much more important symptom than in those where it occurs without obvious implication of the functions of nutrition." The treatment in these cases must be directed to the restoration of the functions of the stomach, bowels, and skin; to the suppression of the habitual excitement under which the kidney labours, and to the confirmation of the health generally.

*Azoturia* is a term applied to a condition where the urine is characterized by a superabundance of urea; another form, according to our author, of *diabetes insipidus.* It will be found, on analysis, that the quantity of urea, in proportion to the solid ingredients, is in these cases very large. The calls to void the water are very urgent: the general symptoms are those connected with loss of strength, feelings of languor, a greater or less degree of emaciation, &c. Cases of diabetes reported to have been cured are considered by Dr. Willis as belonging likewise to this head. The functions of the kidney are regarded as being increased, the organ being in a state of erythism, requiring general and local bleeding, succeeded by blisters and remedies acting beneficially on the general health. The following directions for analysis of the foregoing conditions of the urine will be very acceptable to many of our readers.

"The general appearance, degree of transparency, disposition to deposit, odour, &c. are to be noted. The specific gravity is then to be ascertained. A given quantity of urine, say 1000 grains, is then to be slowly evaporated, at a temperature not exceeding 160 or 200 degrees of Fahrenheit, till it ceases to lose weight. The quantity of extract being ascertained by weight, the proportion of the solid matters to the water will become equally known. The extract is next to be digested with strong boiling alcohol (sp. gr. ·833), which will take up the urea and salts (the lactates) which are soluble in alcohol. What is dissolved is to be poured off; what remains is to be washed once or twice with a little fresh-boiling alcohol, which is to be added to the first. The alcoholic solution is then to be reduced by slow evaporation to the consistence of extract, or till it ceases to lose weight, and its quantity ascertained. The saline mass, which was insoluble in alcohol, is to be treated with distilled water at 60° F. two or three times; and the different solutions being added together, and evaporated to dryness, the quantity of saline ingredients—the soluble chlorides, and alkaline phosphates and sulphates,—will be discovered. The insoluble matters, consisting principally of the earthy phosphates which remain on the filter, being dried, are to be weighed. They are afterwards to be digested with caustic potash; and, being dried and weighed again, the quantity of mucus or other animal matter will be estimated by the loss of weight, if any, which is sustained. This is as much as is required for medical purposes." (p. 25.)

The disease commonly called ischuria renalis receives from Dr. Willis the name *anuria.* The pathology of the disease is so obscure that it is not surprising that the author has nothing new to communicate concerning it. There is, however, a symptomatic form of the disease, called by Schönlein *urodialysis neonatorum*, which Dr. Willis has occasionally met with. The infants thus affected void very small quantities at a time of extremely high-coloured urine, which stains the linen of a deep reddish colour. It seems to be passed with great pain, and scalds the surfaces over which it runs, exciting inflammation in the mucous lining of the bladder and urethra; as appears by the increased quantity of mucus whice soon passes away with the urine. A more or less febrile condition accompanies this state. The skin is generally irritable, and occupied by eruptions; and, wherever two surfaces come in contact, they almost certainly inflame, and pour out a thin, sharp, fetid fluid. The eruption of the skin occasionally appears in the form of small psydracious pustules, which break, and give rise to superficial and often troublesome sores; especially in the groins, axillæ, folds of the neck, &c.: and a very analogous condition, described by Schönlein as *urodialysis senum*, occurs among persons in the decline of life.

With regard to the histories of vicarious discharges, which are said to have been observed in some instances of idiopathic ischuria renalis, Dr. Willis exercises a very desirable scepticism. All cases the evidence of which rests only on the authority of the patient, we should agree with the author in rejecting; but there are some recorded instances, such, for example, as that mentioned by Dr. Hastings, and referred to in the volume before us, which we cannot believe would ever have been published if there remained in the mind of the narrator a shadow of doubt of their correctness. A fair case for the possibility of such an occurrence may be made out. The characteristic property of urine mainly depends upon the urea, and it is not pretended that the fluid said to have been thus vicariously discharged possessed all the properties of urine. Now, there is no doubt of the existence of urea in the blood in these cases of anuria. We know that bile circulating in the system escapes freely by the kidneys; and we have seen expectoration, in a case of jaundice complicated with pneumonia, possessing all the appearances of bile. With these somewhat analogous facts, and others of a similar nature which might easily be collected, we should be indisposed entirely to deny the possibility of various recorded cases of vicarious discharge of urinous fluid from very unusual channels.

The sedimentary urine, in which the deposit consists of lithic acid and the lithates, is termed by our author *Lithuria.* The condition of the system in which these deposits occur is now tolerably well understood, although there are some prejudices existing concerning it, to which Dr. Willis has very appropriately alluded. We refer to his remarks upon dyspepsia as a cause of the superabundant deposits of lithic acid. Our own observations entirely accord with those of Dr. Willis, and long ago convinced us of the impropriety of so frequently referring this affection to dyspepsia.

"Undoubtedly the digestive organs are simultaneously affected along with every form of general constitutional disturbance, as also with the particular derangements of each and every one of the separate systems of organs which make up the body; but, because they suffer, and because their implication is forced in a peculiar manner on our notice, and because we mostly apply our remedies through the channels they afford us, they are not therefore the causes of these disorders." (p. 71.)

The above quotation is capable of a far more extended application than to the diseases which we are at present considering; and we believe that there is nothing more likely to impede the progress of our acquaintance with these diseases, or to cripple our means of a radical system of cure, than the habit of looking at them all through the digestive system. We have been disposed to regard the various sediments containing lithic acid as more simple than further analysis has shown them to be. Dr. Willis has given the results of the experiments of Vigla and Quevenne, which we subjoin:

" *Red crystalline sediment*, composed of lithic acid and the colouring matter of the urine. The crystals are rhomboidal prisms, which, in the field of the microscope, appear under the form of pretty regular lozenges, generally of a beautiful topaz-yellow colour singly.

" *Lateritious, red, or reddish brown sediment*, composed of lithate of ammonia; purpurate of ammonia and soda; colouring matter of the urine, and occasionally an admixture of the earthy phosphates, (Prout;) lithic acid, combined with the colouring matter of the urine, (Vigla.)

" *Yellowish sediment*, composed of lithate of ammonia, essentially; a little lithate of soda, colouring matter of the urine, more or less of the earthy phosphates, (Prout;) lithic acid combined with less of the colouring matter of the urine than in the preceding form of deposit, (Vigla.)

" *Pink sediment*, composed of lithate of ammonia; purpurate of ammonia, (Prout;) almost wholly of lithic acid; some lithate of soda; an animal matter and a little phosphate of lime, (Vigla and Quevenne.)" (p. 58-9.)

Some objections are offered to the opinion of Dr. Prout and others, that the immediate cause of the precipitation of lithic acid is the presence of a free acid in the urine. It is said that lithic acid, separated from its alkaline base by a free acid, is thrown down in an amorphous, and not in a distinctly crystalline form; that there are great doubts as to the acid by which it is supposed that this precipitation is effected. The muriatic, supposed by Dr. Prout to be the efficient acid in this precipitation, is only known to exist in the urine in combination with a base; the phosphoric retains the lime in solution, and the lactic is the least efficient of all the acids in causing precipitation of lithic acid from its state of solution in the urine: but Dr. Willis does not attempt a chemical explanation of the phenomena in question. There is nothing particularly novel under the head of treatment.

To many of our readers the mode of analyzing lithic urine and its deposits will be very acceptable. A known quantity (1000 grains) of the urine, after it has stood several hours, is to be poured on bibulous paper, and the fluid separated from the solid parts. Add to another portion a few drops of muriatic acid, to ascertain if it contain any additional lithic acid. The precipitation of the lithic acid may always be immediately secured by drying well and warming gently the glass vessel into which the urine about to be examined is poured, or by rubbing the inner surface of the vessel containing the urine, after a few drops of acid have been added to it, with a rod of wood. The colour of the deposits is best seen whilst they are still wet upon the filter. We have already shown how to ascertain the proportion of solid and fluid ingredients. To examine the nature of the deposits, boil a known quantity of any of them with distilled water, by which any alkaline lithates will be dissolved. Evaporate the solution to dryness, and add to the extract a few drops of nitric acid: this dissolves the lithates and lithic acid, with effervescence. The nitric-acid solution being dried with a gentle heat, the pink colour characteristic of purpuric acid will be evolved; and this tint will immediately pass into a bright purple on the addition of a little caustic ammonia. By this means we detect lithic acid. Another portion of the extract being mixed with some caustic lime, if it contains ammonia, this will be discovered by the smell, or by approaching a rod dipped in muriatic acid to the mixture. Another portion of the extract must be burned over the flame of a lamp, in a platinum spoon, and the residue tested for alkaline reaction. The nature of the alkali, whether potass, soda, or lime, is discovered by urging the residue for a moment in the flame of a blowpipe. If it be soda, the outer flame will be coloured yellow. Potass and lime are distinguished by the solubility of the former in distilled water; or, should the lime exist in a caustic state, when it becomes an oxide and then soluble, by a current of carbonic acid gas or a few drops of a solution of oxalic acid. The presence of the earthy salts,—the ammonia-phosphate of magnesia, and the phosphate of lime,—is known by the insolubility of the deposit in boiling distilled water, and by its ready solubility in dilute acid, from which the earthy bases are precipitated on the addition of an alkali. The relative proportion of the differently soluble lithates and the insoluble phosphates may be ascertained by digesting a known weight of the mixed precipitate in a sufficient quantity of attenuated caustic potass or soda, which takes up the lithates, and leaves the earthy phosphates behind.

In the author's remarks on phosphatic urine we find nothing to detain us, except a remark on a condition of the urine which has once or twice led Dr. Willis himself into error, and which, we believe, has been frequently mistaken by others.

"The milky look of the phosphatic urine at the time it is voided in some cases, and the fact of its letting fall a flocculent precipitate when exposed to the boiling temperature, are circumstances which have often led to the supposition that it contained albumen. The mistake is at once detected by adding a drop of the muriatic or nitric acids, by which the precipitate is immediately dissolved, and the fluid rendered completely transparent." (p. 95.)

We must pass over chapters iv. v. and vi., as, although they are full of curious and interesting subjects, industriously collected and very carefully and ingeniously commented on, they are of less practical interest than some other parts of the work. We must make exception, however, to our author's remarks on albuminous urine in certain dropsies. He does not appear to have alluded especially to this condition of the urine as associated with a uniformly low specific gravity. That the urine may be albuminous in dropsy is saying no more than may be said of the urine in health: but our own observation, and we have had some considerable opportunities of noticing the circumstance, would lead us to coincide with those who maintain that where, with dropsy, there is associated an albuminous condition of the urine, the fluid never indicating a density of more than 1.015, but frequently one much below this, there exists a condition of the kidney which will sooner or later terminate in disorganization. It requires a series of years,—more, perhaps, than have yet elapsed since the subject has been so much discussed,—to follow up some slight cases of anasarca, with the above condition of the urine, to their termination; but because, either accidentally or as a result of treatment, the dropsical symptoms have disappeared, we do not therefore consider that the diseased condition has entirely ceased. Let the urine of such an individual be frequently examined after he has recovered a certain degree of health, and we doubt if it will be ever found of the same specific gravity as that of a healthy individual. The kidneys appear to have lost a part of their function; they pass not unfrequently a fair quantity of urine, but far below its proper quality; and slight sources of irritation will restore the albuminous condition of the fluid and the effusion into the cellular tissue. We think that Dr. Willis is disposed to regard as of too little importance, in a diagnostic point of view, the above condition of the urine.

We regret that we have no space for more than a reference to, and a recommendation of, the sections on Oleo-albuminous Urine and on Hæmaturia.

*Melituria* is the term applied by Dr. Willis to *diabetes mellitus.* In reference to the frequency of this disease, it is mentioned by Dr. B. G. Babington, that "his father, the late Dr. Babington, when in very full practice, had found him opportunities of seeing as many as *twenty-three cases of the disease at one time*, in every one of which the sugary state of the urine was ascertained." There are some remarks on the pathology of the disease, which are interesting as facts, but which do not appear to us to throw much light upon its obscurities. To these we shall shortly allude. We do not mention as among the facts above alluded to that Dr. Willis is disposed to believe, with Frank, in the existence of a diabetic virus; an animal poison, which, either introduced into the system or engendered within it, converts food of all kinds into sugar, the sugar circulating in the blood, and producing all the evil effects characteristic of the disease. The theory of animal poisons, although deficient in proof with respect to some diseases, is supported by so much analogy that it often appears to us one of the most satisfactory cities of refuge to which our ignorance may resort. By Ambrosioni, first of all, and afterwards by Mr. Charles Maitland, sugar was detected in the blood of a patient labouring under meliturìa. Mr. M'Gregor, of Glasgow, has detected sugar, in a certain quantity, in the healthy stomach, avoiding all sources of fallacy from food taken into that organ; he has likewise found it in the stomach, mixed with the saliva and with the fæces of diabetic patients; the perspiration has been observed to possess a sweet taste, and the cuticular scales to be similarly characterized in cases of this disease. Taking these facts, together with such reasonings as our knowledge of animal poisons allows us to employ, it is stated that we are in a condition to do more than speculate on the nature of this interesting disease; that a key is afforded to the right understanding of the whole phenomena of meliturìa; and that derangement of the digestive functions is here unquestionably the head and front of the offence; that this derangement is of a peculiar kind, consisting in a disposition of the stomach to form sugar from food. The healthy stomach forms sugar to a limited extent: it is formed to excess by the stomach in meliturìa, and the sugar is not, as in the healthy state, decomposed, but enters the circulation; the symptoms all being explicable on the supposition of a foreign substance circulating in the blood. The derangement of the kidney, it is further supposed, is purely secondary, and is a means of purifying the system from the sugar. The precise nature of the gastric affection, it is admitted, is a question of difficult solution. Founded on this theory of the disease is the following indication of treatment: "*Could we*," says Dr. Willis, "*discover any means of preventing the stomach from forming sugar, we should, I believe, succeed in curing the disease.*"

We have given the above sketch of the pathology of melituria but without being able to regard it, or the indication of treatment founded upon it, as correct. The facts are very interesting, but by no means necessarily lead to the conclusions drawn from them. It is certain that the earliest symptoms of melituria are not known, that consequently from symptoms there is no key to the organ or system of organs which are first of all functionally affected. Dr. Willis supposes the existence of a virus. It has been shown that sugar exists in the blood, in the stomach, in the saliva, in the fæces, in the perspiration. The question then arises, as to where the disease (the first effects of the virus, if such is supposed to exist,) commences. The sugary condition of all the secretions is doubtless dependent on the state of the blood, and to us there appears to be as much reason to regard the blood as primarily affected as the stomach. With the present amount of our knowledge, we confess the inutility of following any further these speculations; but we are induced to state them, because we do not regard it as at all certain that Dr. Willis has (as above mentioned) discovered, any more than his predecessors, the right indication for treatment in melituria. The remarks made by the author upon dyspepsia, as a condition on which a lithic state of the urine is by no means dependent, we think are fully applicable to diabetes mellitus, and it only surprises us that he has not also been struck with their applicability. From Dr. Willis's remarks on the various remedies employed in diabetes, we quote the following, on magnesia, the effect of which he considers as somewhat *specific* in the disease.

"Mr. Benjamin Phillips lately prescribed magnesia, upon two occasions, to the same individual labouring under melituria in an early stage, and in each instance with success: the sugar disappeared from the urine, the thirst and all the other symptoms of the disease were immediately relieved and speedily vanished. I had lately under my care at the Infirmary for Children, a child about five years of age, with the symptoms of melituria well marked; the urine was copious, of high sp. gr. (1.033,) passed spontaneously into fermentation, yielding abundant bubbles of carbonic acid gas for several days, and acquired something of a vinous ordour. The constitutional symptoms were also those of the disease; there was wasting, thirst, shrivelled skin, &c. The magnesia in half-drachm doses in mint-water three times a day, three grains of Dover's powder at night, castor oil in quantity sufficient to keep the bowels open, and a regulated diet consisting principally of eggs and meat, had the effect of relieving all the symptoms in the course of four days: the thirst was appeased, and the urine was reported as nearly natural in quantity." (p. 231.) Unfortunately, Dr. Willis now lost sight of the case.

Mr. M'Gregor's mode of detecting urea in diabetic urine is this: "He destroys the sugar in a given quantity of urine by fermentation with yeast, evaporates to dryness in a steam bath, treats the residue with hot strong alcohol, filters, evaporates the solution to dryness, and obtains the urea in a crystalline form, not pure indeed, but sufficiently so for all practical purposes."

Fermentation with yeast is the most delicate and available test for sugar; half a grain of sugar in two ounces of urine being easily detected by it. The test may be thus employed: "The yeast should be introduced into the bottom of a phial, and a given weight of urine having been gently poured in, so as not to disturb it, the mixture is to be placed in a temperature from 70° to 80° F. At the end of forty-eight hours, the fermentation will have ceased; and the degree of attenuation undergone by the fluid will afford an index to the quantity of alcohol formed, otherwise, to the quantity of sugar destroyed."

There is one advantage possessed by a physician over a surgeon, in treating the subject of renal and vesical calculi. Cutting is no part of his province, and he consequently endeavours, as far as possible, to prove its inexpediency. The opposite error is one against which he must guard himself. He requires, perhaps, to be cautioned, lest with his deluges of solvents, introduced into the system and into the bladder, he fails of the proposed object, and leaves his patient in a more unfavorable condition than he found him. The last chapter of the first part of Dr. Willis's book is devoted to renal and vesical calculi. As it is too extensive for us to notice entirely, we shall select from it the evidence which exists in favour of other than surgical methods of curing stone in the bladder. We think that Dr. Willis has made out a very fair case, and we feel somewhat surprised, with him, that, with the evidence which exists on the subject, more attempts have not been made to effect the solution of calculus. He remarks that, "if so excellent a chemist as Mr. Brande, in England, has almost ridiculed the notion of our ever effecting the solution of a stone in the bladder, Fourcroy and Vauquelin, in France, and Berzelius, in Sweden, have held this feasible, and have encouraged us to persevere; if M. Civiale has declared such a thing impossible, his countrymen MM. Robiquet and Ch. Petit, in Paris, and Sir B. Brodie have, in different ways, performed the feat." We select from a numerous catalogue of them, the following facts. Cloquet found that a calculus of lithic acid subjected to the action of *pure water* for five hours a day, during a month actually *lost a line and a half in its diameter*. So much for simple diluents. The alkaline bicarbonates contained in the waters of Vichy have been found very efficient in their action upon various kinds of calculi. The water contains a large proportion of free carbonic acid, and very nearly a drachm and a half of bicarbonate of soda in each thousand drachms of the menstruum. At a temperature of 97° F. the Vichy water speedily dissolved lithic-acid gravel. The half of a lithic acid calculus, weighing one ounce one drachm and thirty-six grains and a half, was placed in a little bag of wire muslin and subjected to the action of the Vichy water during 151 hours. Dried and weighed after this, the calculus was found reduced to two drachms, fifty-two grains. In a similar bag, calculi of phosphates were disintegrated, and their particles washed through the meshes of the muslin. Very little change was produced upon a calculus of oxalate of lime. A piece of an ammoniaco-magnesian phosphatic calculus, weighing 31.50 gr., immersed for eighteen days in the water, was found reduced to 17.25 gr., having lost 45.23 per cent. From the ascertained action of the Vichy water upon various calculi, it is concluded, that it will "attack something like nineteen-twentieths of all the known varieties of urinary concretion." Such are among the facts observed out of the body. Cases recorded by Dr. Petit, of Vichy, lead to the conclusion that similar solution or disintegration has followed their use as copious diluents, internally. But the action of these and similar waters must not be regarded as simply diluent: they act much more rapidly on the lithic acid, lithate of ammonia, and triple phosphate of ammonia and magnesia, than pure water. Besides increasing the quantity of the urine, they alter its chemical constitution; from acid, it becomes neutral and then alkaline; from high coloured, it becomes pale; from having deposited copiously, it becomes limpid and transparent. The remarks which have been made upon the action of waters containing in solution alkalies and alkaline carbonates, apply very much to artificial solutions of the same. The repeated influence of such medicines is well enough known; and few are not acquainted with celebrated instances of their effects, as well as with the objections which have been made to their supposed solvent powers, and the explanations which have been given of the benefit which has often attended their use. It is important to bear in mind, that it is not in a caustic state that the influence of the alkalies is most evident; that weak solutions of the alkaline bicarbonates act as certainly and almost as quickly upon urinary concretions of lithic acid as solutions of the caustic alkalies of equal strength. Calculi of the triple phosphate of lime are not *dissolved* by solutions of the alkaline bicarbonates; but they are *disintegrated* and rapidly reduced to powder by their agency, and others which cannot be so acted upon are of unfrequent occurrence.

The subject of the *solution of stone by means of injections into the bladder* is one of very great interest; and the statements and facts of Dr. Willis on this head are such as deserve the most attentive consideration. We have seen the effect of the Vichy waters upon calculi external to the body, and it is not difficult to subject calculi within the bladder to the continued action of solvents. We do not see that Dr. Willis has alluded to a very admirable method of passing a current of fluid through the bladder. It is practised by M. Civiale in Paris in some diseases of the bladder, and probably by others. A bucket is suspended from the ceiling above the bed of the patient. A flexible tube passes from it and communicates with one partition of a double catheter. The fluid escaping from this bucket traverses one division of the catheter, washes the bladder, and escapes afterwards through the other partition. We shall be excused, if, although not of recent date, we shortly allude to some of the facts mentioned by Dr. Willis, in illustration of the solvent effects of injections of the bladder. Dr. Rutherford, in 1753, relates the case of a patient, with a large calculus in the bladder. Under the united influence of alkaline medicines by the mouth, and injections of lime-water by the urethra, the stone was reduced to a mere nucleus, and eventually disappeared, being undiscoverable by a sound. Berzelius on this subject says, "The attempts that have hitherto been made to dissolve calculus in the bladder have not answered expectations: but I am intimately persuaded that they have not been repeated often enough to allow of those accidental circumstances which are never to be foreseen, but which always occur (and oppose success in new courses) from becoming perfectly known, and either guarded against or vanquished." MM. Magendie and Amussat brought away, by means of a thin injection slightly acidulated with sulphuric acid, a large quantity of calculous detritus. Sir B. Brodie's case is known to most of our readers, in which, by the use of a diluted nitric acid injection, the solution of phosphatic calculi was effected, until they were so reduced in size as to escape from the urethra during an effort to make water. "I am thus," says Dr. Willis, "enabled to adduce three instances, connected unquestionably with as many different diatheses, in which, under the influence of injections into the bladder, combined in the one case with the exhibition of alkaline medicines by the mouth, (which was probably a part of the treatment necessary to success,) uncomplicated and without such assistance in the other two, in all of which complete success attended the persevering efforts that were made to afford relief." Certainly, the cases adduced by Dr. Willis are such as to encourage attempts to dissolve calculi in the bladder. We are disposed to think that one of the reasons why this method has not been attempted more frequently, is, that it requires great patience on the part both of the surgeon and the sufferer; and if we could supply this quality of patience, combined with most assiduous attention, as easily as we can recommend it, we think that other diseases besides calculus, which generally lead to corporeal mutilation, might be regarded as of tolerably certain cure.

Our notice of Dr. Willis's work must here terminate. It is one which we have redd, and trust again to read, with profit. The history of discovery is succinctly given; cases, curious and important, illustrative of the various subjects, have been selected from many new sources, as well as detailed from the author's own experience; chemical analyses, not too elaborate, have been afforded which will be most convenient to those who wish to investigate the qualities of the urine in disease; the importance of attending to this secretion in order to a proper understanding of disease is strongly insisted upon: in short, a book has been composed, which was much required, and which we can conscientiously and confidently recommend as likely to be useful to all classes of practitioners.

